# Supplementary material for: Changes in the Ultrastructure of Staphylococcus aureus Treated with Cationic Peptides and Chlorhexidine
Source: Microorganisms. 2020 Dec 14;8(12):1991. doi: 10.3390/microorganisms8121991 (PMC7764955; doi:10.3390/microorganisms8121991)
Supplement: Supplementary file 1 [file microorganisms-08-01991-s001.pdf]

Supplementary Materials

for article

Changes in the Ultrastructure of *Staphylococcus aureus* Treated with Cationic Peptides and Chlorhexidine

Alina Grigor'eva, Alevtina Bardasheva, Anastasiya Tupitsyna, Nariman Amirkhanov, Nina Tikunova, Dmitrii Pyshnyi and Elena Ryabchikova

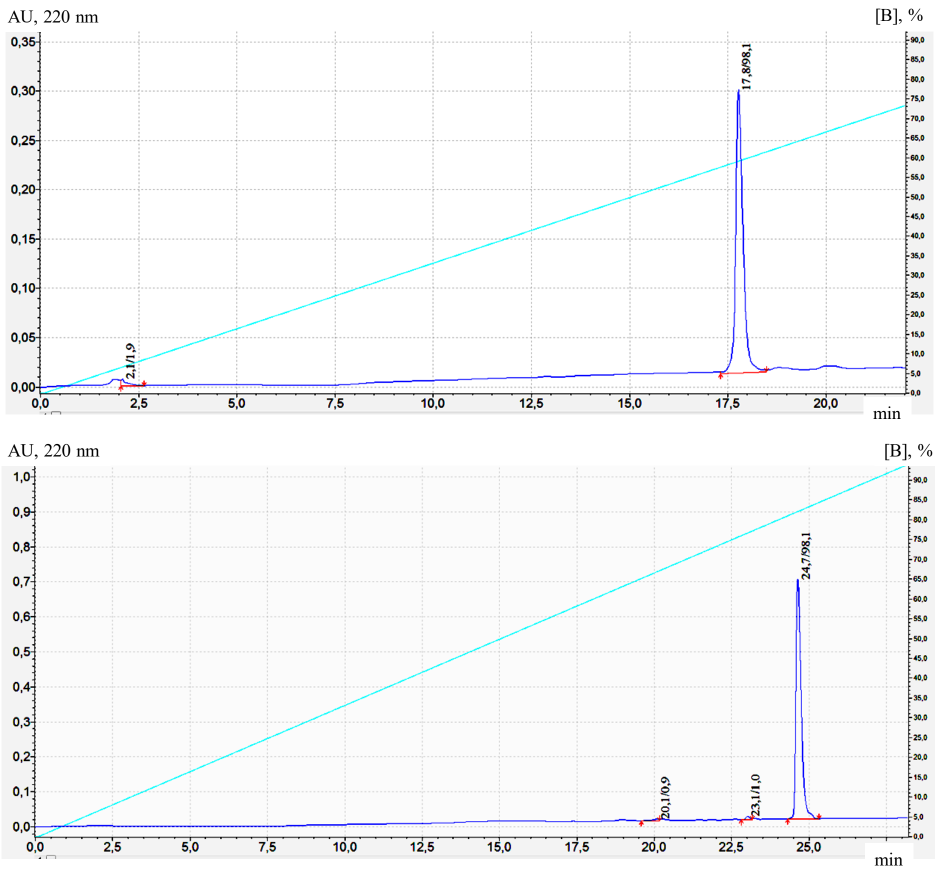

Figure S1. RP-HPLC of the purified R9F2 peptide (upper image) and (KFF)3K peptide (bottom image) on a Gemini column (5 μm NX-C18, 110A, 4.6 × 250 mm; Phenomenex Inc, United States) balanced with a 0.1% TFA solution in a linear gradient of acetonitrile concentration (0–50%) for 30 min at a flow rate of 1 mL/min. The UV detection was performed at a wavelength of 220. Buffer B = 50% CH_3CN in 0.1% TFA.

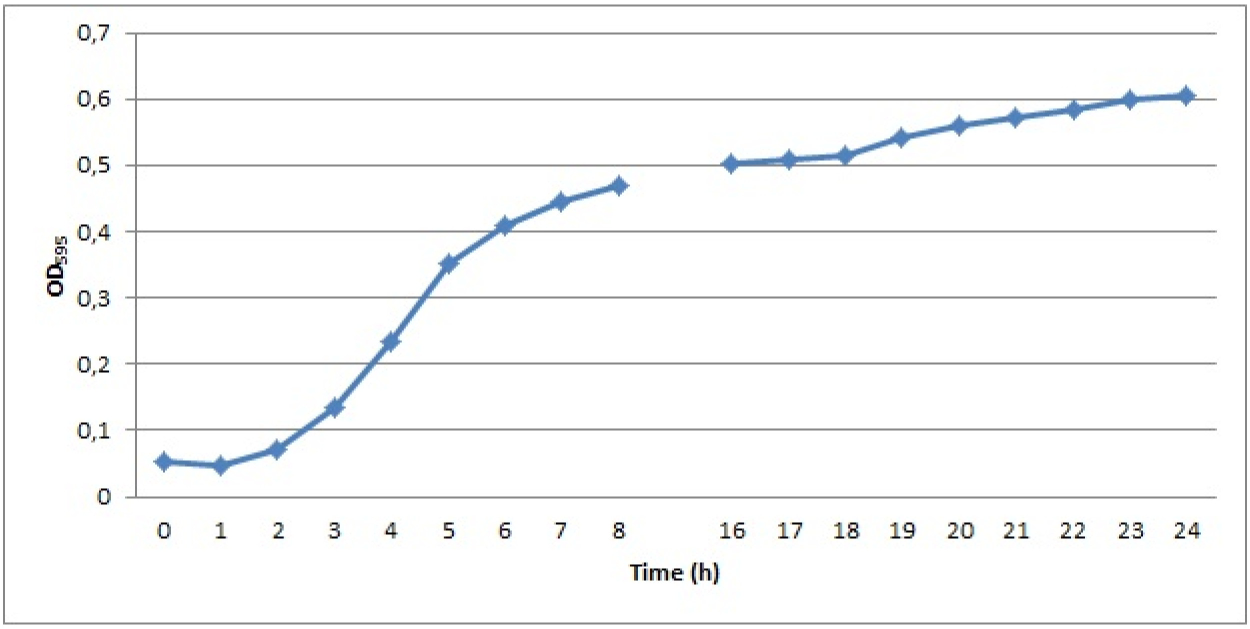

Figure S2. Growth curve of S. aureus in LB-culture medium at 37 °C.

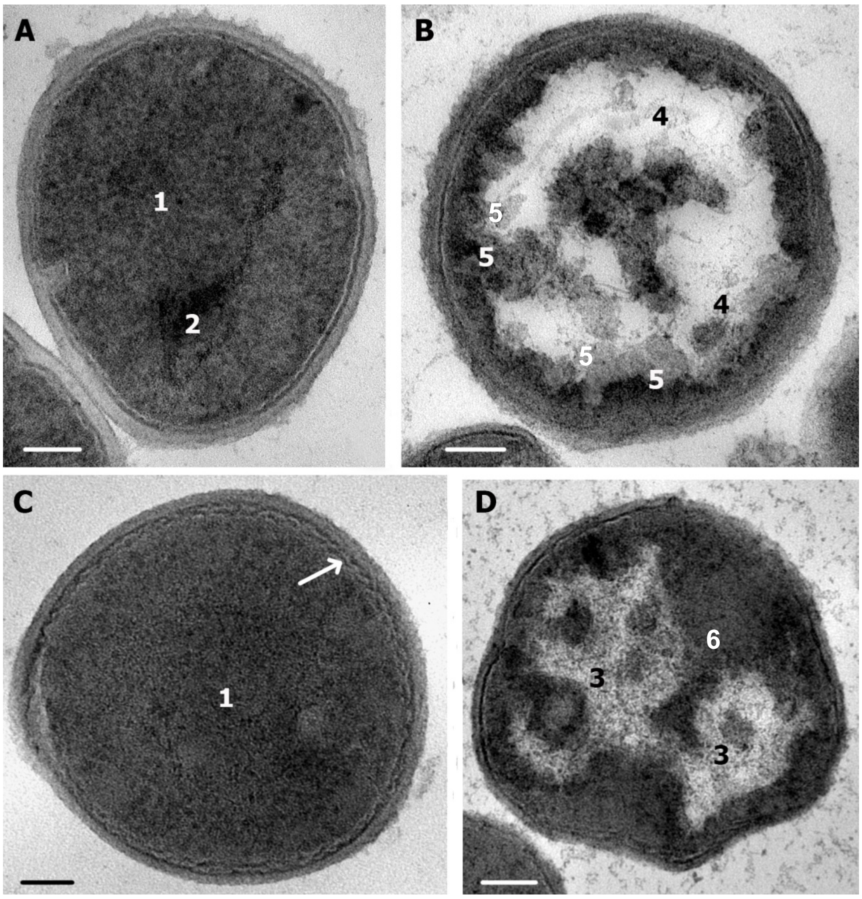

Figure S3. Ultrastructure of *S.aureus* cells incubated with R9F2 peptide for 1 min (**A**) and 15 min (**B**); and with (KFF)3K peptide for 1 min (**C**) and 15 min (**D**). 1 – cytoplasm, 2 – nucleoid, 3 – area of damaged cytoplasm of low electron density; 4 – "empty" areas in cytoplasm; 5 - amorphous clumps, 6 –massif of electron dense amorphous material. White arrow shows cell membrane with pronounced undulation. TEM of ultrathin sections. Scale bars correspond to 100 nm.

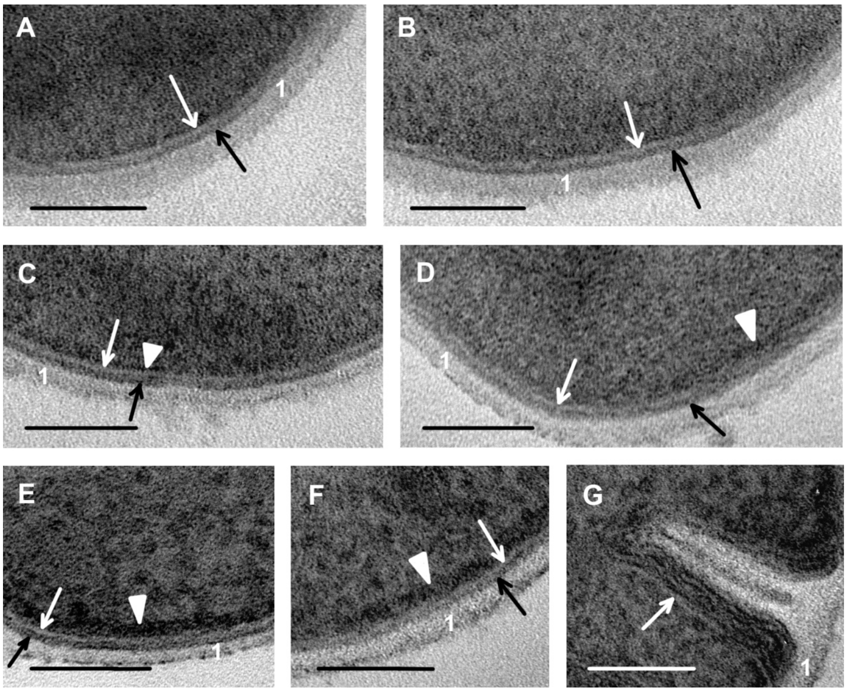

Figure S4. Ultrastructure of *S.aureus* envelope incubated with chlorhexidine for 15 min (A,B), 30 min (C), 60 min (D) and 120 min (F-H). 1 – cell wall, black arrows show intermediate layer, white thin arrows show cell membrane, and white thick arrows show a layer under the cell membrane formed by electron-dense particles of the cytoplasm. TEM of ultrathin sections. Scale bars correspond to 100 nm.
